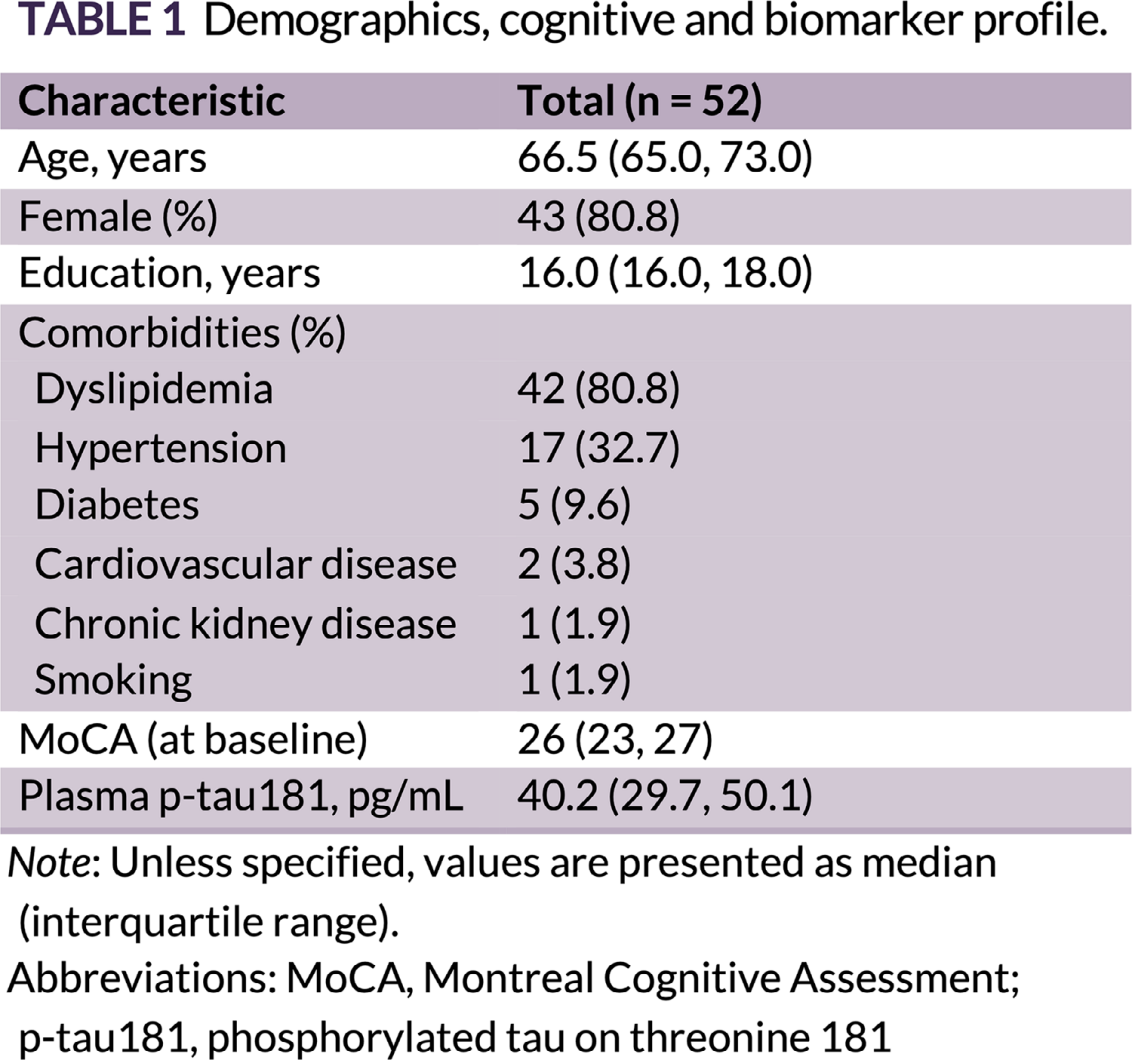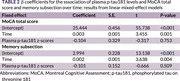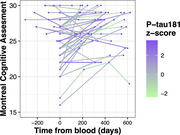# Association Between Baseline Plasma p‐tau181 Levels and Cognitive Trajectory in Healthy Geriatric Populations

**DOI:** 10.1002/alz.092823

**Published:** 2025-01-09

**Authors:** Rapas Samalapa, Poosanu Thanapornsangsuth, Pasin Hemachudha, Watayuth Luechaipanit, Thanaporn Haethaisong, Adipa Chongsuksantikul, Gann Boonyaprapatsara, Chaipat Chunharas, Thiravat Hemachudha, Kearkiat Praditpornsilpa

**Affiliations:** ^1^ Geriatric Excellence Centre, King Chulalongkorn Memorial Hospital, The Thai Red Cross Society, Bangkok Thailand; ^2^ King Chulalongkorn Memorial Hospital The Thai Red Cross Society, Bangkok Thailand; ^3^ Thai Red Cross Emerging Infectious Diseases Health Science Centre, King Chulalongkorn Memorial Hospital, Bangkok Thailand; ^4^ Faculty of Medicine, Chulalongkorn University, Bangkok Thailand; ^5^ Chula Neuroscience Center, King Chulalongkorn Memorial Hospital, Bangkok Thailand; ^6^ Elderly Health Care Center, Queen Savang Vadhana Memorial Hospital, Sriracha, Chonburi Thailand; ^7^ Chula Neuroscience Centre, Bangkok, Bangkok Thailand; ^8^ Chersery Home International, Bangkok Thailand; ^9^ Cognitive, Clinical and Computational Neuroscience (CCCN) Center of Excellence, Chulalongkorn University, Bangkok Thailand; ^10^ Division of Neurology, Department of Medicine, Faculty of Medicine, Chulalongkorn University, Bangkok Thailand

## Abstract

**Background:**

Dementia is a critical concern in the aging population, and understanding biomarkers associated with cognitive trajectories can provide valuable insights to improve diagnosis and intervention strategies.

**Method:**

We conducted our study at a geriatric check‐up clinic at King Chulalongkorn Memorial Hospital, focusing on a healthy cohort of older adults aged 60 or older without dementia. This study included patients with available plasma p‐tau181 levels (Quanterix, Simoa) concurrent with cognitive evaluations no more than one year before the blood test, as well as at least one cognitive test administered after the blood test. The data collection period extended from March 2022 to January 2024. Cognitive evaluation was conducted using the Montreal Cognitive Assessment (MoCA), with the total score and subsection MoCA scores serving as outcome measures. The data were analyzed using linear mixed‐effects models, incorporating time from the blood test and plasma p‐tau181 level as fixed effects, with individual subjects considered as random effects.

**Result:**

Among 52 healthy participants, the median age was 66.5 years (IQR: 65‐73), the median education was 16 years, and 80.8% were female. Baseline assessments showed a median MoCA score of 26 (IQR: 23‐27) (Table 1). The fixed‐effects model revealed a negative association between plasma p‐tau181 levels and MoCA total score (coefficient = ‐0.104) as well as memory subsection scores (coefficient = ‐0.101), although the findings were not statistically significant. In contrast, a significant positive association was observed between time and MoCA total score (coefficient = 0.003, p = 0.001) and memory subsection (coefficient = 0.002, p = 0.004) (Table 2).

**Conclusion:**

The observed overall cognitive improvement over time in our study might imply a learning effect. Notably, among participants showing a positive correlation between time and cognitive performance, there is a trend towards lower cognitive function in those with elevated plasma p‐tau181. The lack of statistical significance in the association with plasma p‐tau181 may be attributed to small sample size and limited study duration. Our study suggests a potential association between plasma p‐tau181 and cognitive trajectories in a healthy geriatric population; however, future investigations with larger cohorts and extended follow‐up durations are necessary.